# RNA Virus Evolution via a Quasispecies-Based Model Reveals a Drug Target with a High Barrier to Resistance

**DOI:** 10.3390/v9110347

**Published:** 2017-11-17

**Authors:** Richard J. Bingham, Eric C. Dykeman, Reidun Twarock

**Affiliations:** 1 Departments of Mathematics, University of York, York YO10 5DD, UK; r.j.bingham@york.ac.uk (R.J.B.); e.c.dykeman@york.ac.uk (E.C.D.); 2 Department of Biology, University of York, York YO10 5DD, UK; 3 York Cross-disciplinary Centre for Systems Analysis, University of York, York YO10 5GE, UK

**Keywords:** viral quasispecies, viral evolution, viral assembly, simulation

## Abstract

The rapid occurrence of therapy-resistant mutant strains provides a challenge for anti-viral therapy. An ideal drug target would be a highly conserved molecular feature in the viral life cycle, such as the packaging signals in the genomes of RNA viruses that encode an instruction manual for their efficient assembly. The ubiquity of this assembly code in RNA viruses, including major human pathogens, suggests that it confers selective advantages. However, their impact on viral evolution cannot be assessed in current models of viral infection that lack molecular details of virus assembly. We introduce here a quasispecies-based model of a viral infection that incorporates structural and mechanistic knowledge of packaging signal function in assembly to construct a phenotype-fitness map, capturing the impact of this RNA code on assembly yield and efficiency. Details of viral replication and assembly inside an infected host cell are coupled with a population model of a viral infection, allowing the occurrence of therapy resistance to be assessed in response to drugs inhibiting packaging signal recognition. Stochastic simulations of viral quasispecies evolution in chronic HCV infection under drug action and/or immune clearance reveal that drugs targeting all RNA signals in the assembly code collectively have a high barrier to drug resistance, even though each packaging signal in isolation has a lower barrier than conventional drugs. This suggests that drugs targeting the RNA signals in the assembly code could be promising routes for exploitation in anti-viral drug design.

## 1. Introduction

Eigen’s seminal quasispecies theory [1] laid the foundation for a systematic study of viral evolution, and underpins our current understanding of the interplay of mutation and selection in shaping the evolutionary outcomes of viral infections [2]. However, many predictions of this theory remain qualitative, because the fitness concepts they are based on are often oversimplified due to a lack of biological detail [3,4]. Whilst it would be difficult to introduce fitness functions that simultaneously cover all contributions to viral fitness, attempts were made previously to construct functions that are predictive about specific aspects of viral evolution. For example, a fitness function based on the evolution of amino acid sequences coding for a simian immunodeficiency virus epitope enabled application of a quantitative model to virus evolution and immune escape, demonstrating that quasispecies theory can form the basis for studying real-world viral pathogens [5].

Our understanding of RNA viruses has changed with the discovery of multiple dispersed sequence-structure motifs called packaging signals (PSs) in the genomes of ssRNA viruses that collectively act as an instruction manual for virus assembly. PSs have affinity for their cognate capsid proteins (CPs). As we have shown previously, these interactions have a significant sequence-specific component in addition to non-specific electrostatic interactions [6]. Contacts between PSs and CPs collectively bias assembly towards the most productive pathways [7], effectively solving a viral equivalent to Levinthal’s Paradox [8]. PSs thus embody a virus assembly code in their genomes [9], in addition to coding for the production of viral gene products. This dual function of the genome has important consequences for viral evolution. Coding constraints related to the production of gene products are typically taken into account in models of viral evolution in the form of synonymous mutations, i.e., mutations preserving the amino acid sequences of the proteins. However, the additional pressures on viral evolution arising from the requirement of simultaneously preserving the multiple dispersed signals of the PS code have not been taken into account previously, so that their impacts on viral evolution and therapy have been overlooked.

We introduce here a quasispecies theory-based model of a viral infection that incorporates details of PS-mediated assembly and viral replication. In particular, we monitor viral genomes (vRNAs) in a viral quasispecies, i.e., an ensemble of genetically related viral RNAs, that are each characterised by their PS distribution, i.e., by their phenotype with respect to virus assembly. We use our model of PS-mediated assembly [8] to compute the number of successfully encapsidated genomes of each phenotype during infection of a host cell. This is coupled with a population model of a viral infection, providing a direct link between the mechanisms underpinning virus assembly inside an infected cell and the evolution of a viral quasispecies and disease progression at the population level. We assess the occurrence of therapy-resistant mutant strains in drug therapy directed against recognition of the multiple dispersed PSs in the assembly code in the example of a chronic infection, comparing the outcomes with drugs targeting virally encoded enzymes. Even though the barriers to drug resistance for each individual signal in the RNA-encoded assembly manual are taken to be lower than for the conventional drugs modelled, their multiple dispersed nature, along with the resultant cooperativity in promoting virus assembly, results in a much higher overall barrier to drug resistance. The predictions suggest that drugs directed against the virus assembly code could be a promising avenue for anti-viral therapy.

## 2. Materials and Methods

**Figure 1:**
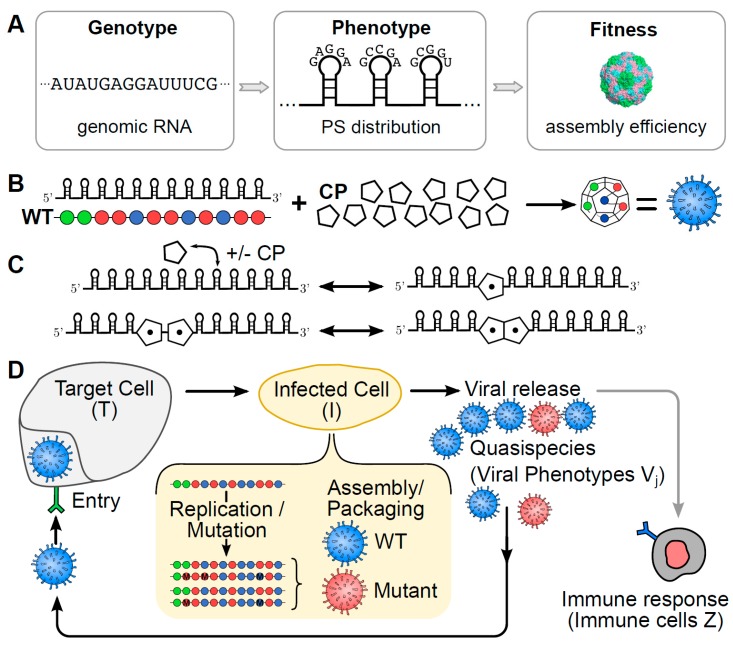
Schematic of the viral infection model. (**A**) The impact of the PS distribution (phenotype) of a vRNA (genotype) on virus assembly efficiency (a proxy for fitness) is captured via a genotype-phenotype-fitness map; (**B**) viral particles are represented as dodecahedral geometries formed from 12 CPs akin to those seen in Picornaviruses. These assemble around vRNAs with 12 PS binding sites, indicated here as strings of beads colour-coded according to their affinity for the CP (with green, blue and red representing strong, intermediate and weak interactions, respectively); (**C**) the interactions between PSs and CPs follow a set of assembly reactions in which CPs are recruited onto the vRNA templates, and neighbouring, bound CPs interact to form the CP-CP contacts required for completion of the capsid shell; (**D**) the different steps of the replication cycle include viral entry into target cells T, replication/mutation and assembly within the infected host cell I, and release of viral particles V into the surrounding medium. Our population level model explicitly contains details of the replication and assembly steps inside the infected cell (shown highlighted in yellow). The immune system removes viral particles dependent on the number of immune cells Z.

Genotype-phenotype-fitness maps [3,5,10] associate fitness values with either viral sequences (genotypes) directly, or with specific viral features (phenotypes) that impact on viral load. In PS-mediated assembly, the PSs constitute the characteristic phenotype of a viral genome (vRNA). PSs in a given vRNA vary around a common recognition motif and secondary structure fold, resulting in differing affinities for their cognate CPs [11,12,13,14,15]. This variation is important for the mechanism of PS-mediated assembly, and determines the percentage of vRNAs that productively assemble into viral particles [8]. As in [8], we base our PS-mediated assembly approach on a dodecahedral model virus, mimicking the geometry of many ssRNA viruses. We assume the existence of 12 PSs per vRNA, the minimal number for a particle with icosahedral symmetry, that interact with CP according to a set of assembly reactions (Figure 1C) to form viral particles. These assembly reactions encapsulate the local rules underpinning the assembly process, akin to those introduced earlier for the protein-only case [16,17]. We therefore characterise the phenotype of a given vRNA via the affinity distribution of its PSs, represented graphically as sequences of beads indicating PS positions in the vRNA, colour-coded according to three affinity bands (see Figure 1). An advantage of using such phenotypes as descriptors of vRNAs is the reduction in computational complexity to a phenotype space of size $3^{12}\approx 5\cdot 10^{5}$, as each of the 12 PS positions can take on one of three distinct affinity bands. This makes identification of the complete fitness landscape computationally feasible over phenotype space. Based on the assembly reactions (Figure 1C), we compute for each viral phenotype the percentage of vRNAs, out of 2000 identical copies, that assemble into infectious virions against a backdrop of cellular competitor RNAs, following our model of packaging signal-mediated assembly [7,8]. This number corresponds to the probability that a vRNA is successfully packaged into a complete viral particle during the assembly step. It is computed at the start of the simulation for each viral phenotype (i.e., for each PS distribution) and retained as fitness values for later implementation in the assembly step of the simulation. This results in an implicit phenotype-fitness map that uses the probability of encapsidation as a proxy for fitness that we will use in an infection model of viral dynamics at the population level (below). The affinity of the PSs for CP in the assembly model used to construct the phenotype-fitness map was chosen as −12 kcal/M, −8 kcal/M and −4 kcal/M, for strong-, intermediate- and weak-affinity PSs respectively. These values are based on MS2 [14,18]. Affinities depend on variations around a core sequence motif, with high-, intermediate- and low-affinity PSs sharing all, most, or only a few sequence-determinants of this motif. All cellular competitor host mRNAs (cRNAs) are assumed to have low affinity for CP. The strength of the CP-CP interactions is −2.5 kcal/M. These parameter choices reflect the range of experimental values observed in an ssRNA bacteriophage, which also undergoes PS-mediated assembly [14].

Our population-level infection model simulates the time evolution of different vRNAs $V_{j}$ (j denoting phenotype) in the viral quasispecies using the standard Gillespie algorithm [19] on the following set of reactions, involving host (target) cells T, cells $I_{j}$ infected by the viral phenotype j, viral phenotypes $V_{j}$, and immune cells Z:

(1) $$T\overset{\lambda }{\to }2T\;\left(\mathrm{Target}\;\mathrm{cell}\;\mathrm{birth}\right)$$

(2) $$T\overset{d_{T}}{\to }0\;\left(\mathrm{Target}\;\mathrm{cell}\;\mathrm{death}\right)$$

(3) $$T+V_{i}\overset{\beta }{\to }I_{i}\;\left(\mathrm{Infection}\;\mathrm{of}\;\mathrm{target}\;\mathrm{cell}\;\mathrm{by}\;\mathrm{phenotype}\;i\right)$$

(4) $$I_{i}\overset{a}{\to }\underset{j}{\sum }k_{ij}V_{j}\;\left(\mathrm{Infected}\;\mathrm{cell}\;\mathrm{death}/\mathrm{lysis}\right)$$

(5) $$I_{i}+Z\overset{p}{\to }Z\;\left(\mathrm{Infected}\;\mathrm{cell}\;\mathrm{removal}\;\mathrm{by}\;\mathrm{immune}\;\mathrm{system}\right)$$

(6) $$V_{i}+Z\overset{u}{\to }Z\;\left(\mathrm{Virion}\;\mathrm{removal}\;\mathrm{by}\;\mathrm{immune}\;\mathrm{system}\right)$$

(7) $$I+Z\overset{c}{\to }I+2Z\;\left(\mathrm{Immune}\;\mathrm{cell}\;\mathrm{birth}\right)$$

(8) $$Z\overset{b}{\to }0\;\left(\mathrm{Immune}\;\mathrm{cell}\;\mathrm{death}\right)$$

Here, λ and $d_{T}$ denote the birth and death rates of target cells respectively, β indicates the infection rate and *a* the death rate (or lysis rate) of infected cells, while *p* and *u* represent the rates at which infected cells and virus respectively are cleared by the immune system. Immune cells are created at rate *c* and die at rate *b*.

The $k_{ij}$ terms in the reaction for the death (lysis) of infected cells correspond to the viral production rate, defining the viral burst size at cell lysis of different phenotypes $V_{i}$. This term is dependent on the parent phenotype $V_{j}$ that has infected the host cell (infected cell $I_{j}$). Its value depends on the replication and assembly process inside the infected cell (see Figure 1D). For a ssRNA virus, this includes error prone replication of the genome into negative and positive strands by the viral polymerase, as well as the synthesis of viral proteins and the formation of viral particles. Here we neglect details of protein synthesis, and instead only model the accumulation of viral copies, which is implemented in our model by repetitive copying of the positive and negative strands available until 2000 positive strands (vRNAs) have been accumulated. For each, we associate its probability of packaging according to its phenotype (i.e., its PS distribution), and thus compute the phenotypes and numbers of fully encapsulated vRNAs at cell burst. When an infected cell lysis event occurs in the discrete equations above, we compute the $k_{ij}$ for that individual infected cell $I_{j}$ as follows:

The ***replication step*** is simulated by creating positive-sense copies from negative-sense templates and vice versa. Starting with the parent phenotype $V_{j}$, polymerase randomly copies any of the positive-sense, or in later rounds also negative-sense templates present in the infected cell, until 2000 positive-sense vRNAs are accumulated. Copying errors result in mutations that are assumed to occur with a fixed per-nucleotide mutation rate of $M_{R}=1/L$ per nucleotide, which equates to, on average, one nucleotide error per genome-copying event, as is typical of Picornaviruses [20]. Since we are working with phenotypes instead of genotypes, PSs are mutated at a rate of $M_{PS}=M_{R}\cdot 0.05/12$ per PS per genome, reflecting the situation where approximately 5% of the genome contains sequence motifs important for PS function.
The ***assembly step*** is simulated by giving each positive-sense vRNA created in the replication step the chance to package based on its phenotype and the associated probability of packaging obtained from our pre-computed phenotype-fitness map. Mimicking in vivo scenarios, our PS-mediated assembly model simulates ssRNA virus assembly against a backdrop of cRNAs. The latter are associated with a uniformly small chance of packaging. Successfully encapsidated cRNAs act as immunogens in our model, stimulating the immune response, as they are indistinguishable from viral particles at the particle exterior. However, although cRNAs are allowed to enter target cells, they do not result in the production of additional viral particles. Following the assembly step, progeny vRNAs and misencapsidated cRNAs that are fully encapsidated are released into the extracellular environment and are added to the total viral load.

We note that the reactions of our discrete model imply a system of ODEs (see Supplementary file S1) at the population dynamics level that is similar to previous population dynamics models of viral infections [21,22]. Our equations differ slightly from those in [21]. Despite these differences, both models result in similar dynamics at the population level. However, unlike continuum models, the discrete stochastic simulation allows us to track large numbers of viral phenotypes while also describing the replication events in individual infected cells, the latter of which is critical for simulating the effects of anti-viral drugs targeting PSs.

Rate constants in the reactions underpinning the viral infection model have been chosen such that the progression of the viral infection reflects experimental results. In particular, they have been adapted to reflect estimates for viral load in 1 ml of blood ($10^{6}-10^{9}$ viral particles) based on data for Hepatitis C virus [23]. We therefore chose the number of viral particles at equilibrium to be $V_{eq}=10^{7}$, and the numbers of target cells, infected cells and immune cells to be $T_{eq}=10^{6}$, $I_{eq}=10^{4}$, and $Z_{eq}=10^{4}$, respectively. Following examples in [20], we specified the death rates of target cells, immune cells and infected cells as $d_{T}=0.1$ cell^−1^ d^−1^, b=0.1 cell^−1^ d^−1^ and a=0.5 cell^−1^ d^−1^, respectively. Equilibrium conditions imply relations between model parameters that determine the birth rate of immune cells as $c=1.5\times 10^{-5}$ cell^−2^ d^−1^. Moreover, the target cell production rate, and the viral and infected cell clearance rates by the immune system depend on the infection rate $\beta =1.0\times 10^{-6}$ virions^−1^ cell^−1^ d^−1^, and are given by $\lambda =5.05\times 10^{6}$ virions^−1^ cell^−1^ d^−1^, $u=0.36\times 10^{-5}$ virions^−1^ cell^−1^ d^−1^, and $p=1.5\times 10^{-2}$ cell^−2^ d^−1^, respectively. These are equivalent to the dimensionless quantities used in [20] ($u=10^{7}$ and $p=10^{5}$) when multiplied by the equilibrium populations of the relevant species (immune cells and virions for u, and immune cells and infected cells for p). Note that variation of the immune clearance rates, p and u at approximately the same ratio does not affect the outcome.

## 3. Results

Since multiple RNA PSs have been discovered in viruses causing chronic infections, such as Hepatitis C virus (HCV) [13], we apply our quasispecies model to this viral system and compare the resulting evolution of the quasispecies when targeted by both conventional drugs and drugs targeting PSs. Experimental results available for a licensed NS5A inhibitor (Daclatasvir; BMS-790052) and the NS5B polymerase inhibitor Sofosbuvir (PSI-7977) are used to develop a model that enables comparison with a (hypothetical) PS-binding drug.

**Figure 2:**
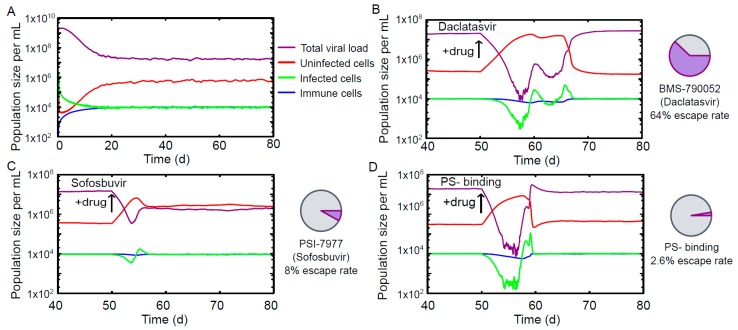
The time course of a chronic infection based on data for Hepatitis C virus. (**A**) Time evolution of viral load, host, infected and immune cells in the drug-free case. 20 days after the initial infection, the population sizes have reached a stable equilibrium corresponding to a viral load of 10^7^/mL. Typical time courses for viral quasispecies that have developed resistance to Daclatasvir (**B**); Sofosbuvir (**C**); and a PS-binding assembly-inhibitor (**D**). The probability of the quasispecies developing therapy resistance is significantly lower for the PS-binding drug. At the drug concentration for which infection of a single cell with the wild-type sequence results in the same number of viral particles at cell burst under treatment with Sofosbuvir and the PS-binding drug (8.07 µM); the drug escape for the PS-binding drug is 2.6%, compared with 8% for Sofosbuvir. A similar comparison with Daclatasvir using a PS-binding drug concentration of 10.30 µM results in a drug escape rate of 1.7% for the PS-binding drug (not shown), compared with 64% for Daclatasvir.

Figure 2A shows a typical profile for a chronic infection in the absence of drug action. Reaction rate constants have been chosen (see Materials & Methods) such that the differential equations have a stable fixed point corresponding to a viral load of $10^{7}$ mL^−1^, consistent with experimental results [23]. Details specific to HCV replication, such as the copying preferences for negative strands over positive ones observed in RNA-dependent RNA polymerases [24], are included explicitly in the reactions describing the replication (Figure 1).

Daclatasvir targets the non-structural protein NS5A, and escape mutations involve mainly the amino acids L31, Q54, and Y93. Quasispecies evolution is modelled on the level of the RNA coding sequence, using the 9 nucleotides coding for this combination of leucine, glutamine and tyrosine as a descriptor of the vRNA, instead of the beads representing PSs similar to [5]. The space of vRNAs thus consists of $4^{9}\approx 10^{5}$ different nucleotide sequences. We consider an infection caused by a founder virus with sequence CUGCAGUAC, the wild type. Mutations are generated for these sequence fragments during our replication step using a mutation rate of $M_{R}=1/L$ per nucleotide. Using experimental values for the replicative fitness of different NS5A phenotypes (see Supplementary Table S1, adapted from Table 1 in [25]), we adapt the numbers of progeny vRNAs produced in the replication step depending on the infecting parent phenotype. Drug resistance is modelled using the experimentally obtained resistance profile for Daclatasvir (see Supplementary Table S1, adapted from Table 1 in [25]), which is implemented in the model by adjusting the numbers of progeny vRNAs generated in the replication step. Similarly, the NS5B-targeting Sofosbuvir has been modelled based on experimental values for replicative fitness and drug resistance (see Supplementary Table S1, adapted from Table 5 in [26]). In this case, six amino acids (T179, S282, M289, I293, M434 and H479) are affected by mutations.

**Figure 3:**
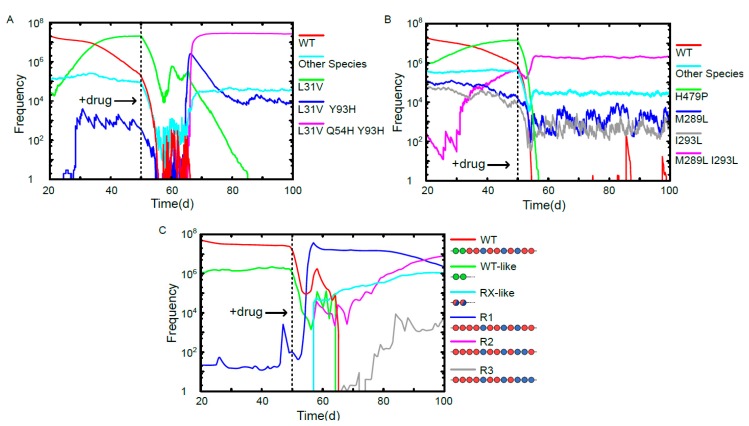
Evolutionary trajectories of vRNAs in the viral quasispecies in a drug escape scenario. (**A**) Time evolution of vRNAs in the viral quasispecies under the conditions of a chronic infection and exposure to Daclatasvir at day 50. WT and mutants L31V, L31V Y93H, L31V Q54H Y93H are shown on an individual basis, and all other species are grouped together. L31V is on the pathway to the therapy-resistant triple mutant L31V Q54H Y93H, which is the dominant species from day 66; (**B**) time evolution of vRNAs in the viral quasispecies under a chronic infection treated with Sofosbuvir at day 50. WT, H479P, M289L, I293L and M289L I293L are shown on an individual basis, and all other species are grouped together. At day 50, the single mutant H479P is dominant, but has poor drug resistance, and is displaced as the dominant vRNA by the more resistant double mutant M289L I293L after day 55; (**C**) the time evolution of vRNAs in the viral quasispecies under chronic infection conditions and exposure to a PS-binding assembly-inhibitor at day 50. WT and the three emergent therapy-resistant vRNAs R1, R2 & R3 are shown individually, while WT-like vRNAs (characterised by having two high-affinity PSs at the 5’ end as in the WT) and R1/2/3-like vRNAs (containing only intermediate and low affinity PSs) are shown as groups. The dominant species following drug exposure is R1, which is then displaced by R2 after day 87. The sequence R3 is present at low frequencies at day 100, but becomes the dominant species from day 190.

The time evolution of characteristic phenotypes in the quasispecies for the scenarios of viral escape are shown in Figure 3. The performances of Daclatasvir and Sofosbuvir are benchmarked against the PS-binding drug. To enable comparison between the existing HCV treatments and the PS binding in the absence of detailed pharmacokinetic information, we chose model parameters so that the susceptible vRNAs are subject to the same knock-down in viral production in each case, thus isolating the effect of drug escape on viral load. This is implemented via differing drug concentrations for the PS-binding drug: 10.30 µM and 8.07 µM for comparison with Daclatasvir and Sofosbuvir, respectively. Minimal barriers to drug escape (one escape mutation) are assumed for each PS in isolation. As shown in Figure 2, we find that under these conditions Daclatasvir and Sofosbuvir have escape rates of 64% and 8%, respectively, over 100 simulations, while the PS-binding drug has only a 2.6% or 1.7% escape rate over 1000 simulations for the lower (8.07 µM) and higher (10.30 µM) drug concentrations, respectively.

## 4. Discussion

Recent technological advances in genome sequencing have resulted in a wealth of experimental data on RNA virus evolution [27,28] that is bringing long-standing open questions regarding viral evolution within reach [3]. Mathematical models can play a key role in addressing these in tandem with experiments. However, the simplified nature of the fitness concepts on which these models are based, lacking key features of viral evolution [5] and mechanistic insights into the life cycles of these viruses, often reduces the scope and predictive power of the modelling. While fitness landscapes covering all aspects contributing to viral fitness are difficult to achieve, there have been a number of successful approaches in which fitness concepts have been adapted to biological processes of interest, such as host entry and adaptation to new host environments [29], replication [30], and immune recognition of viral epitopes [6]. Here, we have constructed a fitness function that captures essential features of RNA virus assembly. It is based on a detailed understanding of the roles of PSs in RNA virus assembly. The increasing number of viruses from distinct families, and infecting different hosts, for which multiple ordered contacts between genomic RNA and capsid have been observed (e.g., [11,31,32,33])—a signature feature of a PS-mediated assembly scenario—suggests that this mechanism may be widespread. All these viruses are amenable to our model.

The model provides a framework for addressing many open questions that rely on a fitness concept linking virus assembly and viral load. For example, it can serve as a basis to study directed misencapsidation for genetic interference via Therapeutic Interfering Particles [34,35], methods of controlling replication fidelity to create attenuated virus vaccines [36], and anti-viral strategies targeting the assembly step. We have focused here on anti-viral therapy and the occurrence of resistant mutant strains, which is one of the key challenges in virology. Increased human mobility and climate change have resulted in the spread of new and emergent viral disease such as Chikungunya virus, yet efficient forms of therapy or prevention are lacking for many of these viral threats, in part due to the rapid occurrence of drug-resistant mutant strains. This is particularly the case for viruses in the group of single-stranded RNA viruses that comprises many important human pathogens ranging in severity from HIV to the common cold, because the mutation rates of their viral genomes are high compared with those in DNA viruses. The discovery of PS-mediated assembly in RNA viruses presents an unexploited therapeutic target with, as is demonstrated here, a number of promising properties. For example, there are indications from studies of Human Parechoviruses that the PS recognition motifs and their contact sites on their cognate CPs are conserved across the entire genus [11], so that drugs targeting these interactions could potentially act simultaneously against different types of a viral pathogen, including those encompassing epitope variants.

As multiple PSs in a viral genome vary around a common recognition motif and act collectively in promoting virus assembly, they are distinct from other types of antiviral drug targets. Our model captures the complex dependencies of viral fitness on mutations in different subgroups of PSs of a given viral RNA, revealing distinctive properties of PS-mediated assembly as a drug target. These advantages, most notably a high barrier to drug resistance, could be a universal feature of therapy against such RNA viruses, including major human pathogens. As therapy should immediately start with as many drugs as clinically possible [37], PS-binding drugs could also play an important part of a combination therapy. This makes PS-binding drugs a promising route for further exploitation in drug design that could open up a step-change in anti-viral therapy.

## Supplementary Materials

The following are available online at www.mdpi.com/1999-4915/9/11/347/s1.
