# Supplementary material for: RNA Virus Evolution via a Quasispecies-Based Model Reveals a Drug Target with a High Barrier to Resistance
Source: Viruses. 2017 Nov 17;9(11):347. doi: 10.3390/v9110347 (PMC5707554; doi:10.3390/v9110347)
Supplement: Supplementary file 1 [file viruses-09-00347-s001.pdf]

Supplementary Materials for: 'RNA virus evolution via a quasispecies-based model reveals a drug target with a high barrier to resistance'

Richard J. Bingham, Eric C. Dykeman & Reidun Twarock

File S1: The ODE model corresponding to the reactions underpinning the simulations

The reactions of our discrete model imply the following system of ODEs at the population dynamics level:

$$\frac{dT}{dt} = \lambda - d_T T - \beta\, T\, V_j$$
$$\frac{dI_j}{dt} = \beta\, T\, V_j - a\, I_j - p\, I_j\, Z$$
$$\frac{dV_j}{dt} = a \sum_l k_{jl}\, I_l - \beta\, T\, V_j - u\, V_j\, Z$$
$$\frac{dZ}{dt} = c\, I_j\, Z - b\, Z.$$

Similar equations have been used previously in population dynamics models of viral infections to investigate the role of immune escape during drug therapy [1, 2]. Note that our equations differ slightly from those in [1] in that there is an additional $\beta\ TV_j$ term in the differential equation for V_j that describes the loss of a virion from the extracellular environment when it enters a target cell, and a matrix k_{jl} that captures the contributions to viruses of phenotype j in the quasispecies from the burst of infected cells I_l. Despite these differences, both models result in similar dynamics at the population level. However, unlike continuum models, the discrete stochastic simulation allows us to track large numbers of viral phenotypes while also describing the replication events in individual infected cells, the latter of which is critical for simulating the effects of anti-viral drugs targeting PSs.

Drug parameters for Sofosbuvir and Daclatasvir

We have considered three drug treatments: the PS-binding drug, and the existing HCV treatments Sofosbuvir and Daclatasvir. While pharmacological information (such as their EC50 values) are available for Sofosbuvir and Daclatasvir, we instead use here the replication and resistance levels for both drugs that have been measured in [5] for Sofosbuvir and [4] for Daclatasvir (see also Table S1), as this is the closest equivalent to the phenotype-fitness map data that we have generated for our PS-binding drug. In order to create a basis for comparison, the PS-binding drug was adjusted such that the same viral load is seen at cell burst, after infection of a single cell with the wild-type sequence as for Sofosbuvir and Daclatasvir. As a result, the fractions of escape mutants within the viral load are directly comparable for the different drug treatments. This results in a drug concentration of $C_D = 8.07\mu$M in comparison with Sofosbuvir, and of $C_D = 10.30\mu$M in comparison with Daclatasvir. Over 1000 runs at these drug concentrations, the PS-binding drug shows a drug escape frequency of 2.6%, respectively 1.7%. These levels of drug escape are well below the corresponding values of 64% and 8% seen for Daclatasvir and Sofosbuvir (see Fig. 2).

We considered the effect of decreasing the binding strength of the PSs with highest affinity for the drug. In the HCV infection model, the levels of drug escape rose to 3.0% (for $K_d = 50$nM) and 4.2% (for $K_d = 100$nM) for the Sofosbuvir comparative concentration ($C_D = 8.07\mu$M) and to 2.5% (for $K_d = 50$nM) and 2.9% (for $K_d = 100$nM) for the Daclatasvir comparative concentration ($C_D = 10.3\mu$M). All these values are below the corresponding values for Daclatasvir and Sofosbuvir. Details on the parameters used in the simulations are collated in Table S1.

Implementation of PS drug strategies

For our hypothetical PS targeting drug, escape mutations can occur through a single mutation at a PS site, but this mutation will come at the cost of a reduced affinity for CP. The drug binding affinities are adapted from previous work [3].

Replication and resistance levels for the Hepatitis C virus replication inhibitors Daclatasvir and Sofosbuvir

Daclatasvir

vRNA	Replication Level (%)	Fold Resistance	Replication vRNAs produced (-drug/+drug)
WT	100	1	2000/20
L31M	99	3	1980/20
L31M Q54N/H	83	3	1643/20
L31M Y93H	70	7,105	1400/1378
L31V	158	28	3160/717
L31V Q54N/H	131	28	2623/593
L31V Y93H	50	14,789	1000/992
Q54N/H	83	1	1660/16
Q54N/H Y93H	22	9	440/36
Y93H	27	24	540/123
L31M Q54N/H Y93H	100	19,000	2000/1976
L31V Q54N/H Y93H	189	19,000	3780/3735

Sofosbuvir

vRNA	Replication Level (%)	Fold Resistance	Replication vRNAs produced (-drug/+drug)
WT	100.0	1.0	2000/300
T179A	89.0	1.1	1780/383
S282T	30.5	2.8	610/248
M289L	99.9	2.9	1998/829
I293L	93.9	1.3	1878/459
M434T	104.7	0.9	2094/385
H479P	126.0	1.0	2520/503
T179A M289L	44.8	2.1	896/305
M289L I293L	150.4	2.5	3008/1143
T179A M289L I293L	95.6	3.5	1912/879
S282T M289L	32.3	6.3	646/388
S282T M289L I293L	45.2	5.6	904/518
T179A S282T I293L	1.8	8.3	36/23
T179A S282T M289L I293L	6.6	9.5	132/91
S282T M289L H479P	72.5	7.4	1450/926
S282T M289L I293L M434T	83.0	7.1	1660/1045
S282T M289L I293L H479P	126.0	8.4	2520/1680
T179A S282T I293L H479P	85.5	9.0	1710/1165
T179A S282T M289L I293L M434T	45.1	10.6	902/645
T179A S282T M289L I293L H479P	38.8	11.8	776/571
T179A S282T M289L H479P	0.26	11.4	4/2

Table S1: The resistance and replication levels for the two Hepatitis C virus replication inhibitors modelled in the simulations. The data for Daclatasvir (BMS-790052, top) and Sofosbuvir (PSI-7977, bottom) is adapted from [4] and [5], respectively. The final column shows the viral replication levels in the drug-free (- drug) case and under drug action (+ drug). Combinations of mutations not found in the literature are derived via multiplication of the corresponding values of the single mutants.

References

[1] Nowak M, May RM (2000) *Virus dynamics: mathematical principles of immunology and virology.* (Oxford University Press, UK).

[2] De Boer RJ (2012) Which of our modeling predictions are robust? *PLoS Comp Biol* 8:1.

[3] Dykeman EC, Stockley PG, Twarock R (2014) Solving a Levinthal's paradox for virus assembly identifies a unique antiviral strategy. *Proc Natl Acad Sci USA* 111:5361.

[4] Fridell RA, et al. (2011) Genotypic and phenotypic analysis of variants resistant to hepatitis C virus nonstructural protein 5A replication complex inhibitor BMS-790052 in Humans: In Vitro and In Vivo Correlations. *Hepatology* 54:1924.

[5] Lam AM, et al. (2012) Genotype and subtype profiling of PSI-7977 as a nucleotide inhibitor of hepatitis C virus. *Antimicrob Agents Chemother* 56:3359.
